# Multi-view image-based behavior classification of wet-dog shake in Kainate rat model

**DOI:** 10.3389/fnbeh.2023.1148549

**Published:** 2023-05-02

**Authors:** Salvador Blanco Negrete, Hirofumi Arai, Kiyohisa Natsume, Tomohiro Shibata

**Affiliations:** Department of Human Intelligence Systems, Graduate School of Life Science and Systems Engineering, Kyushu Institute of Technology, Kitakyushu, Japan

**Keywords:** wet-dog shake, rat, deep learning, animal behavior, multi-view, behavior classification

## Abstract

The wet-dog shake behavior (WDS) is a short-duration behavior relevant to the study of various animal disease models, including acute seizures, morphine abstinence, and nicotine withdrawal. However, no animal behavior detection system has included WDS. In this work, we present a multi-view animal behavior detection system based on image classification and use it to detect rats’ WDS behavior. Our system uses a novel time-multi-view fusion scheme that does not rely on artificial features (feature engineering) and is flexible to adapt to other animals and behaviors. It can use one or more views for higher accuracy. We tested our framework to classify WDS behavior in rats and compared the results using different amounts of cameras. Our results show that the use of additional views increases the performance of WDS behavioral classification. With three cameras, we achieved a precision of 0.91 and a recall of 0.86. Our multi-view animal behavior detection system represents the first system capable of detecting WDS and has potential applications in various animal disease models.

## 1. Introduction

Animal behavior analysis plays an essential role in pre-clinical models investigating the causes of human diseases. Hence, automating and finding new insights into behaviors is fundamental to accelerating research. Machine vision techniques are widely recognized as effective instruments for automating behavior analysis. However, most systems are limited to one view, in mice, usually from the top. Nevertheless, a single view cannot be used to detect all behaviors. Although a top view is suitable for analyzing the displacement of a rat in a cage, it may not be suitable for observing behaviors that involve detailed limb movements.

Wet-dog shake behavior consists of a rapid oscillation of the body (Dickerson et al., 2012). In rats, it is analyzed by recording experiments from a side view and then reviewed manually. Still, it can be challenging even for humans, especially when the rat faces opposite to the camera. The behavior is unpredictable and has a short duration, which makes it easy to miss. In our experiments, it only accounts for 0.38% of the time despite using an over-WDS disease model. In this study, we analyze WDS in Kainate (KA) treated rats, where WDS is one of the behaviors used to evaluate seizure progress during treatment (Lévesque and Avoli, 2013). WDS is also present in morphine abstinence, and nicotine withdrawal, among other studies (Wei, 1973; Suemaru et al., 2001; Vuralli et al., 2019; Shahzadi et al., 2022; Yunusoglu et al., 2022). Studying WDS behavior could help us understand animal model diseases and their human equivalents. Despite numerous systems developed for animal behavior detection in recent years, none of them can detect WDS behavior.

Live Mouse Tracker is a popular real-time behavior analysis system for mice, but a single camera from the top, and feature engineering make it challenging to adapt to other animals (de Chaumont et al., 2019). Moseq is an unsupervised behavior identification system. It relies on a 3D deep sensor, it is not user-friendly, and the code is not open source (Wiltschko et al., 2015). Another popular machine learning system for quantifying animal behavior is DeepLabCut, which is capable of posture detection and tracking but is not designed explicitly for behavior classification (Mathis et al., 2018). SimBA is a toolkit for behavior classification. However, it uses pose estimation, which requires annotating user-defined key points in addition to the target behavior (Ro et al., 2020). Several commercial behavior analysis systems, such as TopScan, HomeCageScan by Cleversys and Ethovision by Noldus, from which some incorporate multiple cameras, including side views, they operate by generating silhouettes using color filtering techniques. Subsequently, body parts are deduced from these silhouettes, and behaviors are identified by analyzing the predicted body parts in terms of their spatiotemporal patterns (Matsumoto et al., 2020). Nevertheless, these are classic techniques, and the systems fail to incorporate contemporary machine learning techniques that could yield better results. Furthermore, these systems offer limited flexibility, and the absence of open-source code prevents community-driven improvements and modifications. Additionally, the cost should be taken into consideration. To date, no system has been capable of detecting WDS behavior.

In this work, we take inspiration from the multi-view human action recognition (MVHAR) field to address the problem of animal behavior recognition, particularly the WDS behavior in rats. There are two main approaches for MVHAR; the first one is to train end-to-end neural networks (Putra et al., 2018, 2022; Wang et al., 2018; Vyas et al., 2020), but the performance is tied to the use of large MVHAR datasets (Weinland et al., 2006; Gkalelis et al., 2009; Wang et al., 2014; Shahroudy et al., 2016; Guo et al., 2022). Equivalent animal datasets are not available, and producing them would be costly. Another approach is extracting features, with skeleton features being the most used (Zhang et al., 2019; Bian et al., 2023), and then using a separate classifier. Extracting skeleton features requires pose estimators that need extensive amounts of labeled data for training (Insafutdinov et al., 2016; Cao et al., 2017), but labeling animal data can be particularly challenging as it requires familiarity with the target animal anatomy to produce correct labels (Labuguen et al., 2021).

To address these challenges, this study presents a multi-view supervised machine learning system for animal behavior classification. We introduce a novel multi-view-time fusion architecture. At its core, it relies on a simple image classifier. Before classification and multi-view-time analysis, we use the same approach for background removal as (Meratwal et al., 2022) which involves first using an object localization network that generates a bounding box. Then, an image patch is taken according to the bounding box to isolate the subject in the image and remove the background. This approach avoids relaying in hand-crafted feature representations like skeletons and reduces the data needed for training. The WDS dataset we created is a novel non-human multi-view activity recognition dataset with a practical application. Our system can detect the WDS events with a precision of 0.91 and a recall of 0.86 using three cameras. Although we limit this study to the WDS behavior, our system could be trained to incorporate user-defined behaviors across different animals and for classification tasks in general. We hope our system is used to reveal unforeseen features of animal behavior.

## 2. Materials and methods

The experiments were conducted in accordance with the Guide for Care and Use of Laboratory Animals at the Graduate School of Life Science and Systems Engineering of the Kyushu Institute of Technology (Sei#2021-003).

### 2.1. Injection of Kainic acid

The experiments were performed with three young rats aged 4–5 weeks and 104.0−152.5 g (Japan SLC Inc., Japan). There were eight to 10 days of adaptation before the experiment. Light (12 h light–12 h dark), humidity (50+−5 %), and temperature (23 + 1°C) were regulated.

We administered 0.05% KA (5 mg/kg) intraperitoneally after anesthesia (2.5% isoflurane) using the repeated low-dose protocol (Hellier et al., 1998). Every hour three times in total. The animals were recorded for 1 h immediately after the third KA injection.

### 2.2. Hardware information

**FIGURE 1:**
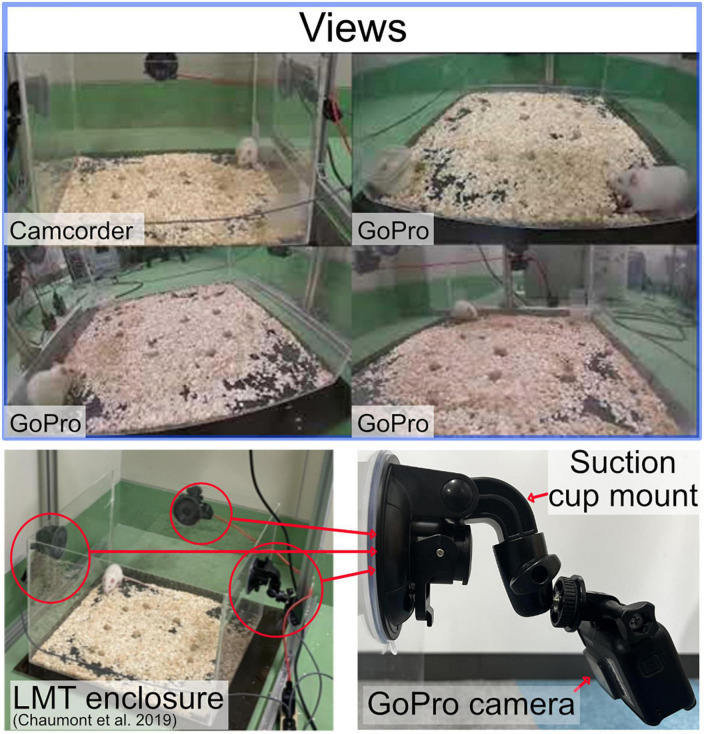
**Top**: the rat being depicted from different perspectives. **Left bottom**: the open source LMT acrylic enclosure. **Bottom right**: GoPro camera attached by a suction cup mount.

We use the live mouse tracker 50 cm × 50 cm plastic cage (de Chaumont et al., 2019). We recorded three experiments with up to four cameras to create a Machine Learning dataset. A Camcorder, a GoPro HERO8 Black, two GoPro HERO7 Silver. A camera was set on each side of the cage. The camcorder was mounted using a tripod, we include a camcorder as this is a common setup for manual labeling (Arai et al., 2022). The GoPro cameras were attached to the plastic enclosure using a suction cup mounted and positioned at the center top of the panel, the angle was adjusted to capture the entire enclosure as Illustrated in Figure 1. The angle and position of the cameras changed slightly between experiments as the cameras were mounted and dismounted between the different experiments. All the cameras were set to a resolution of 1080p at 30 fps. The GoPro HERO8 contains three digital lenses; we used the wide digital lens. The videos were synchronized using Apple Final Cut Pro Multicam editing workflow. The proposed study was conducted using the cloud service Google Colab with a Tesla V100-SXM2−16GB GPU graphics card and an Intel (R) Xeon (R) CPU @ 2.20 GHz hardware configuration.

### 2.3. Data collection

The dataset for machine learning was created using three 1 h recordings after the third KA injection. Two recordings were used for the training dataset. Four cameras were used in the first recording and three in the second. One experiment recorded using three cameras was used for the validation dataset.

#### 2.3.1. Target behavior wet-dog shake

**FIGURE 2:**
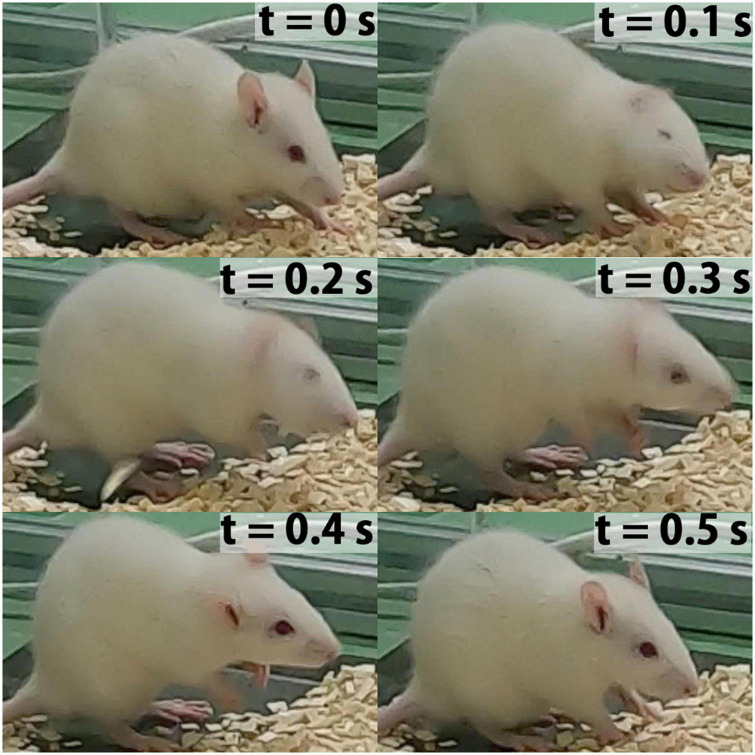
Time-lapse sample frame sequence of WDS behavior recorded in this study (*t* = 0 s previous to the start WDS behavior, *t* = 0.1–0.4 s WDS behavior, *t* = 0.5 s ending of WDS behavior). This behavior is evocative of the movement seen in dogs.

The wet-dog shake behavior occurs naturally as a spontaneous behavior, described as a rapid oscillation of the body as illustrated in Figure 2 (Dickerson et al., 2012). WDS as a naturally occurring behavior is rare and mostly zero when recorded for short periods (Sperk et al., 1985) thus, we only consider KA-treated rats for this study. The KA rat model is an over-WDS model. Soon after the administration of KA, rats experience an unusually high amount of WDS for approximately 1 h until class IV and/or class V seizures appear (Racine, 1972; Hellier and Dudek, 2005; Sharma et al., 2008). In the three experiments used for this study, the animals experienced 149, 220, and 49 WDS events during the hour of high WDS activity after KA administration. The duration mean is 0.33 s with a standard deviation of 0.11 s.

#### 2.3.2. Wet-dog shake event annotation

**FIGURE 3:**
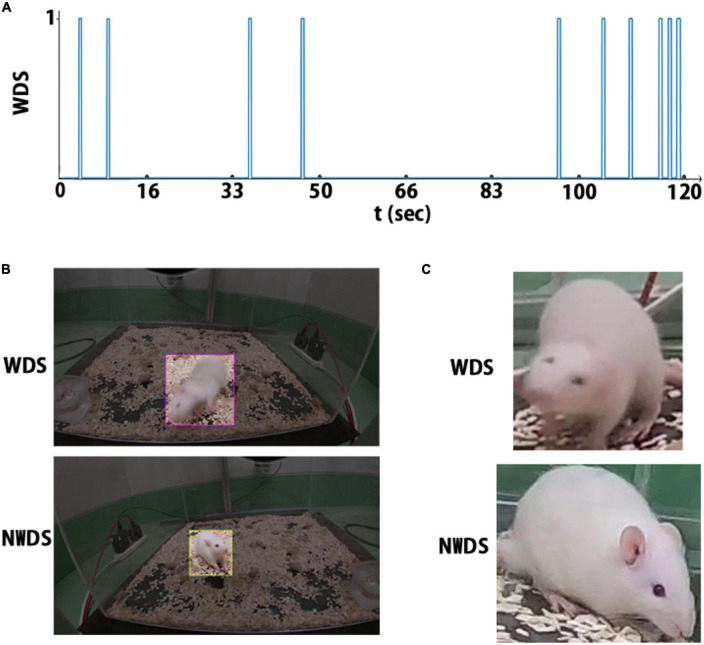
There are three annotation types: **(A)** the rat behavior is labeled at each timestamp. **(B)** Label for object detection, region of interest, and behavior class. **(C)** cropped image and its corresponding behavior class label.

For each WDS event, the frames corresponding to that event are annotated as wet-dog shake (WDS); otherwise, the frames are annotated as no wet-dog shake (NWDS). Figure 3A depicts the event annotations in a 2 min sample, with the horizontal axis corresponding to time and a spike representing a WDS event.

#### 2.3.3. Object localization dataset

For object localization, it is necessary to indicate the animal’s position. The Region of Interest (ROI) describes the animal’s location, a square defined by its width, height, and position (x, y coordinates). Since the video recordings contain more than a million frames, only a subsample was annotated. Frames were selected by extracting the image features utilizing a pre-trained convolutional neural network, specifically MobileNetV2, which was trained on the extensive ImageNet dataset. The extracted features consisted of a 1,280-dimensional feature vector. To simplify the data representation, we reduced the dimensions to 100 principal components using principal component analysis (PCA). Finally, we implemented K-means clustering (Hartigan and Wong, 1979). We used the cloud services Roboflow and Labelbox for annotating the images.

Initially, we labeled 744 frames, 672 for training and 72 for validation. The label includes the WDS event annotation and the ROI. The dataset is balanced, as we selected a similar number of images for each class (WDS and NWDS). Although not necessary for object localization, we added frames where the network failed in the classification task. The final dataset contains 1.5 k images for training. Figure 3B illustrates two example annotations for object localization.

#### 2.3.4. Image classification dataset

We run our entire dataset through the Object Detection Network and take the ROI predictions as ground truth. Then, we crop the images according to the predicted ROI to remove the background. We select all the frames that contain the WDS behavior and then randomly select a similar amount of NWDS behavior images to maintain a balanced dataset. This image classification dataset contains 25,544 frames, 24,920 for training, and 624 for validation. Although this approach gives, for the most part, correct labels, it is not perfect, as the ROI predictions are sometimes inaccurate. Figure 3C shows two examples of annotations, one showing WDS behavior and another NWDS behavior.

### 2.4. Neural networks

**FIGURE 4:**
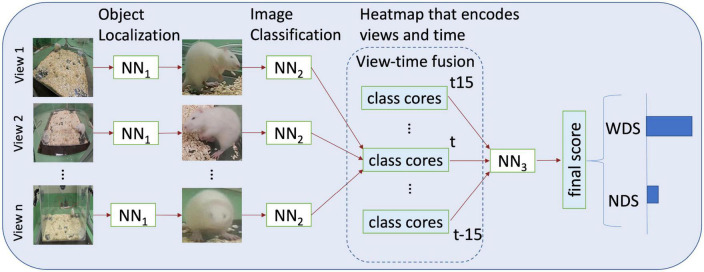
An overview of the method used and the different stages. NN1 object localization; NN2 image classification; NN3 predicts the final score from a feature map encoding time and the multi-views.

For our framework, we use three networks, as illustrated in Figure 4. The first network NN1, is for object localization, the second network NN2, is for image classification and the third network NN3, is to predict the final score from a feature map that encodes the multiple views as well as time. We use TensorFlow (Abadi et al., 2016) on the Google Colab platform, which allows running everything on the cloud if a web browser is available.

#### 2.4.1. Object localization network

We use a Single Shot MultiBox Detector (SSD) (Liu et al., 2016) approach, the fastest meta-network architecture, while maintaining a high accuracy score (Huang et al., 2017). The main advantage of SSD is that it consists of a single feed-forward convolutional network with three stages. Although it can produce ROI and class scores, we only use this network to produce the ROI. Separating the object localization task enables training with fewer labeled data and makes it independent of a particular behavior. The first stage of the object localization network, feature extraction, involves convolutional layers that generate feature maps that encode useful semantic features at different scales and channels. The second stage, the detection head stage, produces ROI and class scores at each feature map scale. The final step is Non-maximum Suppression (NMS), which eliminates repeated predictions. We use the MobileNetV2 (Sandler et al., 2018) for the feature extraction stage, which is also optimized for speed and runs with low hardware requirements, such as smartphones. We use the TensorFlow 2 Detection Model Zoo API (Huang et al., 2017). The network uses pertained weights on the COCO 2017 dataset (Lin et al., 2014). We use it to run on a per-frame base, predicting the rat’s location.

#### 2.4.2. Image classification network

Image classification is described as assigning the label from *k* categories to the image *x*.


(1) f:x→{1,…,k}

Here we train a CNN for image classification; two classes are predicted, WDS and NWDS behavior. We use the WDS classification dataset previously described. As the backbone of the classifier, we use MobileNetV2 (Sandler et al., 2018). Employing a separate classifier allows using a higher picture resolution to make predictions, as the cropped image is created directly from the image with the original resolution.

#### 2.4.3. Multi-view and time series analysis

To accommodate for the multiple views, we modify equation (1), were *x*_*V*_ represents a set of images according to the number of views *n*_*V*_.


(2) $$f:X_{V}\to \left\{1,\ldots ,k\right\}|X_{V}\,\left\{x_{1},x_{2},\ldots ,x_{n_{V}}\right\}$$

Our method uses a score fusion multi-view image classification algorithm. Previous multi-view classification systems that use score fusion employ an aggregation function to predict the final label (Seeland and Mäder, 2021). In contrast, we train a separate network, NN3, that predicts the final score; additionally, to incorporate time analysis, NN2 generates predictions of *X*_*V*_ from *t*−*n*…*t* + *n* as follows:


(3) $$f:X_{V}^{t}\to \left\{1,,k\right\}|X_{V}^{t}=\left\{\begin{array}{ccccc} x_{1}^{t-n} & & x_{1}^{t} & & x_{1}^{t+n}\\ x_{2}^{t-n} & \cdots & x_{2}^{t} & \ldots & x_{2}^{t+n}\\ & & \vdots & & \vdots \\ x_{n_{v}}^{t-n} & & x_{n_{v}}^{t} & & x_{n_{v}}^{t+n} \end{array}\right\}$$

**FIGURE 5:**
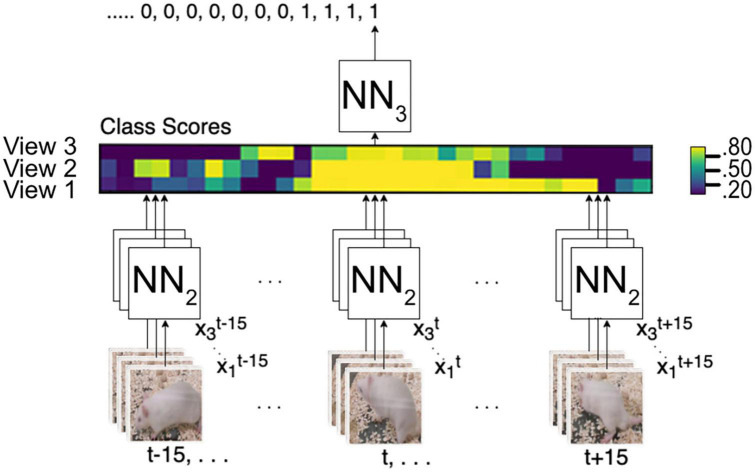
Multi-view integration and time series analysis framework. NN2 analyses all frames from t–15 to t15 from all camera angles to create a vector map from class scores. NN3 generates the final prediction from the vector map.

The output of NN2 is a vector of class scores, as illustrated in Figure 5, and then NN3 predicts the final score. This approach allows NN3 to use multi-view-temporal information. Additionally, with this strategy, only NN3 needs to be trained if we want to adapt to a new environment, add or remove cameras, or change the time window.

**FIGURE 6:**
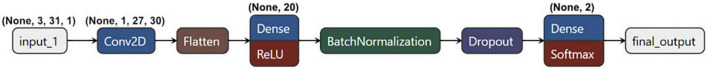
Diagram of the NN3 model architecture, which consists of a Conv2D layer, a flatten layer, a Dense layer and ReLU activation, along with Batch Normalization and Dropout layers.

The NN3 model architecture as illustrated in Figure 6 begins with an input layer, followed by a Conv2D layer with 30 filters and a kernel size of (3, 5). The output is flattened and passed through a Dense layer with 20 units and ReLU activation. Batch Normalization and Dropout layers are employed to improve generalization. Finally, a Dense output layer with 2 units with softmax activation provides classification probabilities for the input data. To further refine and eliminate gaps in the results obtained from the network, we utilize one median and one minimum filter.

## 3. Results

### 3.1. Object detection

The dataset for object detection contains 1572 images, 1500 are used for training, and 72 for validation. In the validation dataset, the network achieves an average Intersection over Union (IoU) of 0.98, meaning the network can correctly localize the animal in the image.

The network we use for object localization also produces a classification score; in the validation dataset, the precision and recall achieved are 0.79 and 0.52, respectively. These results are not enough for our purposes, considering that the dataset used for validation is balanced, which is not the case in the practical application. Therefore, this network is only used to predict the ROI. There are two possible reasons for the low performance in the classification task. The first is the low resolution of the cropped area used to predict the class, and the second is that the network may need more training data.

### 3.2. Image classification

**FIGURE 7:**
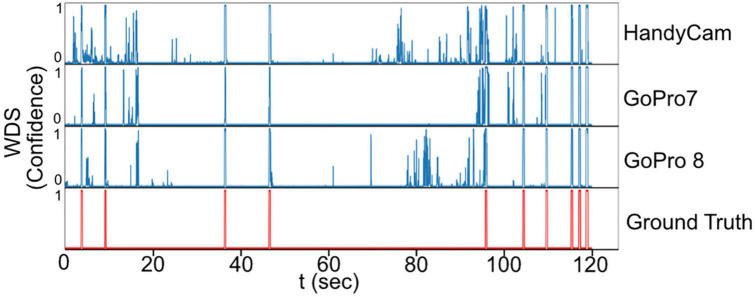
Raw classification prediction in a 2 min video sample. Numerous false positives are detected.

The dataset for image classification consists of 25,549 pictures. For training, 24,920 images are used for training and 629 for validation. The network achieves 92% accuracy in the validation set. Although 92% seems high, in practice, 8% error produces numerous false positives; this is especially problematic as the WDS behavior occupies only 0.38% of the time in our experiments. This point is illustrated in Figure 7, showing the per-frame predictions across the different cameras where many false positives are detected. Although image classification has been used successfully in video classification. In our case, it is not enough, as we only have a few frames to determine the behavior, and the network needs to detect the start and the end of the WDS behavior. So only using image classification alone is not enough.

The image classification results in Table 1 show the correlation between the predictions in different cameras and the ground truth from a 2 min video sample. The camera with the highest score is obtained by the GoPro8 (Pearson’s *r* = 0.81).

**TABLE 1:** Correlation analysis for comparison between different cameras and human labeling.

	Human	GP7	GP8	HC
Human	1.00	0.80	0.81	0.77
GP7	0.80	1.00	0.70	0.68
GP8	0.81	0.70	1.00	0.70
HC	0.77	0.68	0.70	1.00

GoPro7 (GP7), GoPro8 (GP8), handy cam (HC).

### 3.3. Multi-view and time series analysis

**FIGURE 8:**
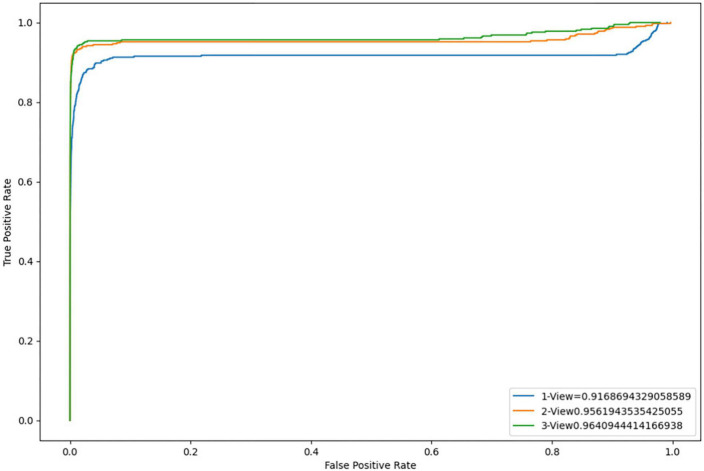
Receiver operating characteristic (ROC) curves for one, two, and three view configurations.

In this section, we test our system in an unseen, 1 h experiment containing 49 WDS events, recorded with three cameras. To evaluate the system, we use recall and precision metrics. This evaluation corresponds to how scientists would use our system to detect the WDS behavior in experiments for the KA rat model. Additionally, we employ Receiver Operating Characteristic (ROC) curves to effectively compare the model’s performance across different configurations, considering varying numbers of views as illustrated in Figure 8.

### 3.4. Performance evaluation

We compare results using different camera configurations to demonstrate whether using multiple views provides performance benefits over using a single view. Table 2 shows the comparison results. We observe that precision is high across all camera configurations, while recall improves when using more views. This confirms the benefits from using additional views.

**TABLE 2:** Comparison of WDS recall and precision metrics with three different camera configurations in the 1 h validation recording.

Views	Precision	Recall
Three	0.91	0.86
Two	0.97	0.65
One	0.90	0.57

In order to further evaluate the performance of our system using different camera configurations, we have included Figure 8, which displays the ROC curves for three different scenarios: 1 view, 2 views, and 3 views. The ROC curves illustrate the trade-off between sensitivity and specificity for each configuration. The results indicate that incorporating additional views improves the overall performance of our system. However, it should be noted that the ROC curves are calculated using raw per-frame data, while in Table 2, the results are counted on a per WDS event basis.

### 3.5. Visual inspection

**FIGURE 9:**
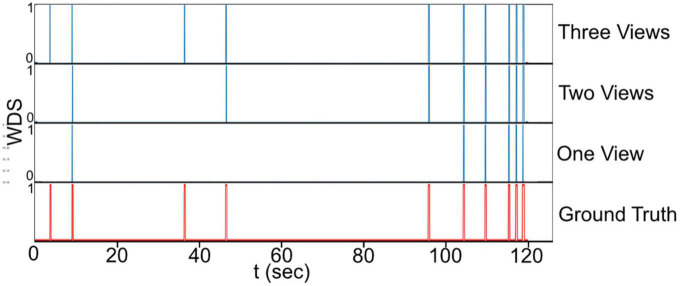
The final prediction of the WDS behavior in a 2 min video sample with different camera configurations.

Figure 9, presents a visual comparison of the final predictions of the multi-view system for WDS behavior in a 2 min video sample extracted from the 1 h validation recording. The 2 min sample is analyzed with different numbers of views. Upon examining the predictions in Figure 9 it becomes evident that the number of WDS events recalled increases with the addition of more cameras while maintaining zero false positives. This improvement in recall can be attributed to the fact that multiple views offer better coverage of the subject’s behavior, reducing the likelihood of missing true instances due to occlusion or orientation. Moreover, the increased information from multiple views also aids the system in discerning between true WDS events and other similar actions, thereby reducing the number of false positives. These observations are consistent with the results in Table 2, where all camera configurations exhibit great precision, and recall rises as the number of cameras grow.

### 3.6. Failure cases

**FIGURE 10:**
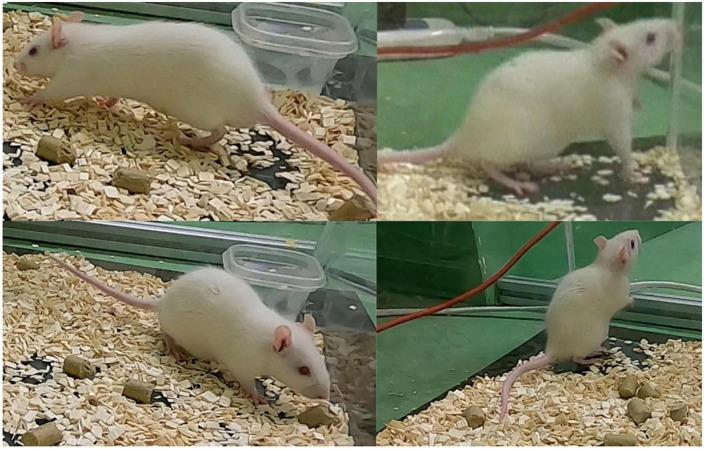
The false positives (four in total) during the 1 h validation experiment where the rat exhibits rearing behavior (two cases) and walking behavior (two cases).

In this section, the failure cases are discussed. Upon careful inspection a total of four false positives were observed during the 1 h validation experiment. Figure 10 illustrates these four failure cases. In two of these instances, the rat exhibited rearing behavior, while in the other two, it was walking.

The false positives in rearing behavior may be attributed to the occasional occurrence of elevation of the forelimb during WDS behavior, which can resemble rearing to some extent as illustrated in Figure 2. To ensure that the model does not consistently confuse rearing behavior with WDS, the first 10 min of the 1 h validation experiment were examined. Seven instances of rearing behavior were identified, none of which were detected as false positives. This observation indicates that the model is generally capable of distinguishing between rearing behavior and WDS.

**FIGURE 11:**
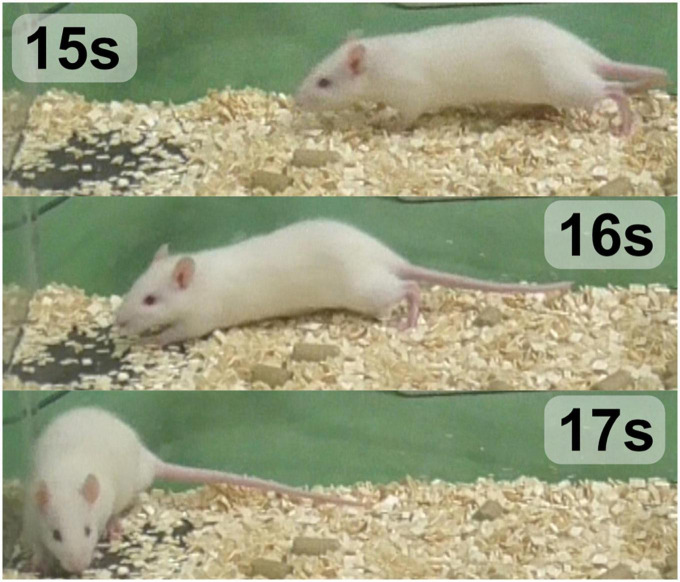
Time-lapse of the rat at the 15–17 s mark, displaying the rat’s walking behavior.

Regarding the false positives involving the rat’s walking, it was noted that the rat moved frequently, but only two instances were incorrectly classified as WDS behavior. In Figure 7, which displays the raw per-frame predictions generated by the image classification network (NN2) across three distinct views, the ground truth is marked in red. Between 15 and 17 s, all the views indicate some probability of WDS, while the rat is actually walking, as illustrated in Figure 11. However, the final prediction by NN3 is successful in filtering out the noise in all view configurations. In this instance, although the WDS probability was high in the raw classification prediction, reaching around 80 percent, for true WDS events, the probability reaches almost 100 percent, showing that the system is capable of distinguishing walking from WDS in most cases.

In the case of false negatives, a clear pattern could be identified to explain the misclassifications. Further investigation and potential refinements to the model may be required to address these instances and improve overall performance.

## 4. Discussion

The human decision-making process frequently depends on the utilization of visual information from a variety of angles. This has inspired the development of machine learning systems that take advantage of multiple views for activity recognition, mainly in humans; still, progress in this field has yet to trickle into animal behavior classification and adapt it to its unique challenges, namely the lack of datasets and the variety of animals across different species used for experiments. In this study, we develop a multi-view animal behavior classification system designed to deal with the major challenges unique to animals. Separating the object localization task allows training with few labeled data, makes it independent to a specific behavior detection task, and avoids degrading the image resolution for classification. Meanwhile, the system can be easily adapted to be used with different amounts of cameras and/or for new environments by only fine-tuning the third network NN3. The system is designed to be easily adapted and used with different animals, behaviors, and other classification tasks while training with little data. In the WDS behavior classification task, our system achieved 0.91 precision and 0.86 recall despite the validation 1 h experiment being recorded on a different date, with a different rat subject, and slightly different camera positioning as the cameras were dismounted between the experiments.

### 4.1. WDS in other rat disease models

Wet-dog shake behavior can be found in various disease models and as a spontaneous behavior (Wei, 1973; Suemaru et al., 2001; Dickerson et al., 2012; Vuralli et al., 2019). The dataset collected in this work only includes the WDS behavior after the KA treatment of rats (Lévesque and Avoli, 2013). Our method should work with other rat disease models where WDS is present, as we could not find studies that suggest WDS locomotion differences between disease models. This also includes WDS as a spontaneous, natural behavior.

### 4.2. Other subjects and classification tasks

Our system could be adapted to be used for other subjects and other classification tasks, including other animals and behaviors. For example, our framework could be used for pure classification tasks. Multi-view image classification is already used for quality control in malting barley (Dolata et al., 2017). With our framework, we could use all the frames from the time barley enters the camera field of view until it is no longer visible while being transported in the conveyor belt.

In the future, we wish to examine the self-grooming behavior. Self-grooming is a complex behavior. In rats, it comprises a series of individual movements that follow a sequence: (0) no grooming, (1) paw licking, (2) nose/face/head grooming, (3) body grooming, (4) leg grooming, and (5) tail/genital grooming (Arai et al., 2022). Rat models of several neuropsychiatric disorders exhibit abnormal self-grooming, each with a distinct phenotype (number, duration, transitions) (Kalueff et al., 2016). For example, in the latent period of the KA rat epilepsy model, rats exhibit an increase in the self-grooming frequency, length, and transition probability 1−2 (paw licking to nose/face/head grooming) suggesting that self-grooming behavior analysis can be used to detect changes of neurons in the dorsolateral striatum as these are related to increases in transition probability in later phases (Arai et al., 2022). Although some systems can detect self-grooming, none analyze individual movements (van den Boom et al., 2017; de Chaumont et al., 2019). These systems use a top view, so discerning the details of self-grooming behavior would be difficult. In contrast, our system could be trained with a better angle view or views that capture self-grooming details and classify each of the five movements involved in self-grooming.

### 4.3. Camera performance and classification

In our study, we observed that the choice of camera plays a role in the classification performance. As seen in Table 1 the GoPro cameras exhibit higher correlations with human labeling. It appears that although the videos were recorded with the same resolution, the GoPro cameras provide higher image quality compared to the camcorder. In Figure 10, the top right image was captured using the camcorder, while the other images were taken with GoPro cameras.

One of the novel aspects of this work is the use of GoPro cameras in behavioral studies. GoPro cameras offer several benefits, such as different lenses, compact size, mounting flexibility, and high-quality image capture. In our experiment, the GoPro cameras were mounted on the acrylic panel using a suction cup mount, which allowed for close and adjustable positioning to the subject. In contrast, the camcorder was positioned on a tripod farther away from the subject.

Future research could explore the impact of camera selection on classification performance in more detail.

## 5. Conclusion

### 5.1. Multi-view behavior classification

Our system integrates multi-view classification and video classification. We offer a novel approach to encode the multiple views and time using a CNN. The system does not rely on feature engineering; it uses object localization and image classification networks as its base, offering great flexibility and making it easy to adapt to other behaviors and animals or any classification task.

Our findings demonstrate the significance of observing animal behavior events from different perspectives.

### 5.2. Wet-dog shake behavior classification

With the increase in scientists using behaviors in the analysis of disease models of animals (Arai et al., 2021, 2022; Arakawa, 2021; Matsumoto et al., 2022), there is a need for automated video analysis systems. This research presents the first system for automatically classifying WDS behavior in rats, a behavior relevant to the study of numerous animal disease models. Additionally, we also provide the first wet-dog shake dataset containing more than 10 h of video, a novel non-human multi-view dataset for activity recognition with a practical application.

## Data availability statement

The raw data supporting the conclusions of this article will be made available by the authors, without undue reservation.

## Ethics statement

This animal study was reviewed and approved by the Graduate School of Life Science and Systems Engineering of the Kyushu Institute of Technology in accordance with the Guide for the Care and Use of Laboratory Animals (Sei#2021-003).

## Author contributions

SN, HA, KN, and TS designed the study. HA carried out the KA injection and recording. HA and SN created the dataset. SN designed, trained, and evaluated the neural network. All authors discussed the results, provided feedback on the writing, and reviewed and approved the final version of the manuscript.
